# Supplementary material for: Intraspecific plant-soil feedbacks alter root traits in a perennial grass
Source: bioRxiv. 2025 Mar 14:2025.03.11.642669. Preprint. [Version 1] doi: 10.1101/2025.03.11.642669 (PMC11952404; doi:10.1101/2025.03.11.642669)
Supplement: Supplement 1 [file media-1.pdf]

Supplementary Figures

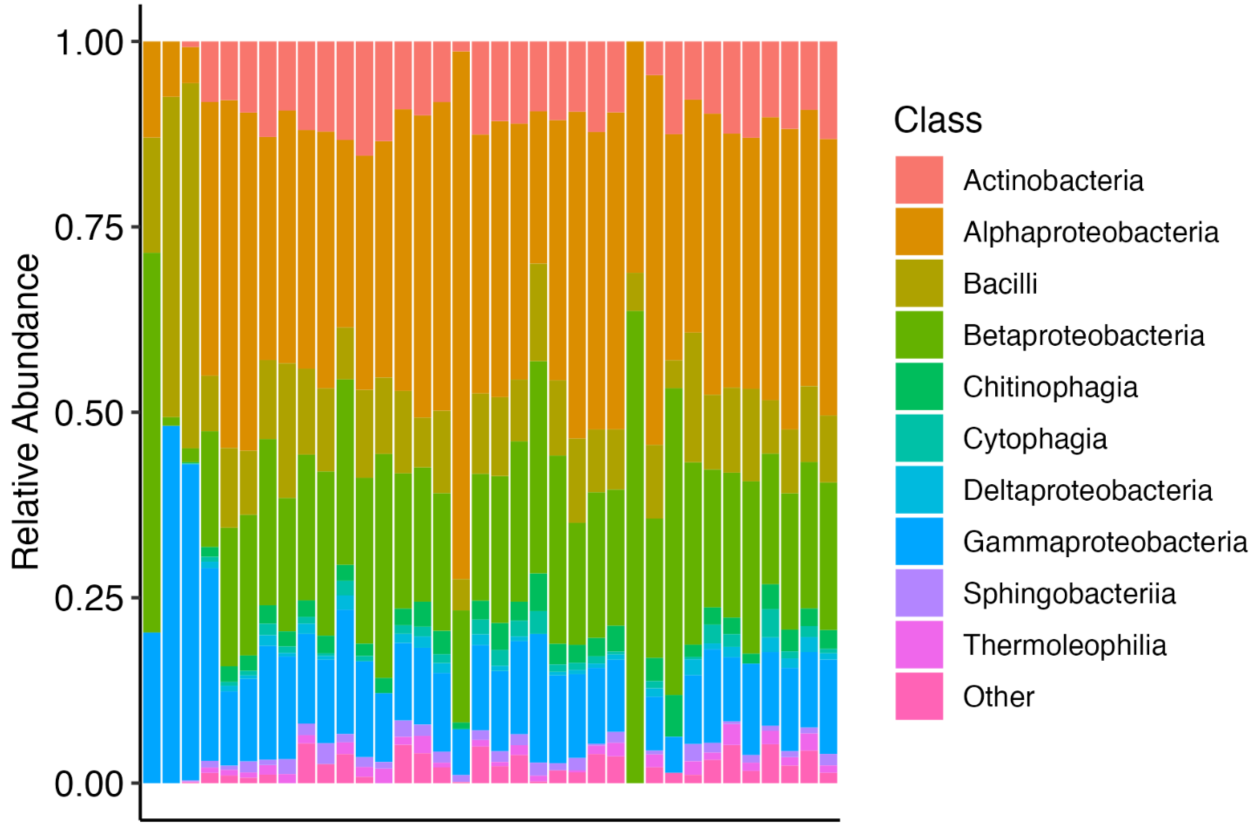

Supplemental Figure 1. Relative abundances of the top ten bacterial classes in the rhizosphere inocula.

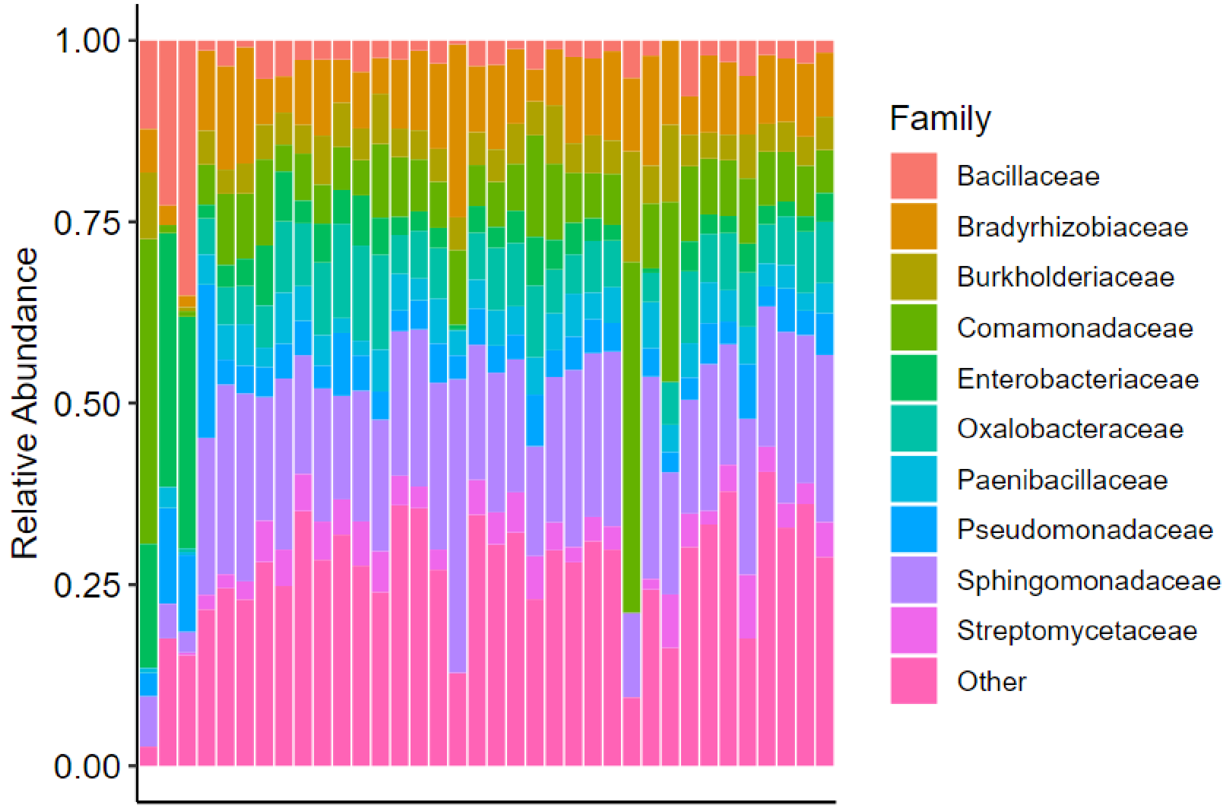

Supplemental figure 2. Relative abundances of the top ten bacterial families in the rhizosphere inocula.

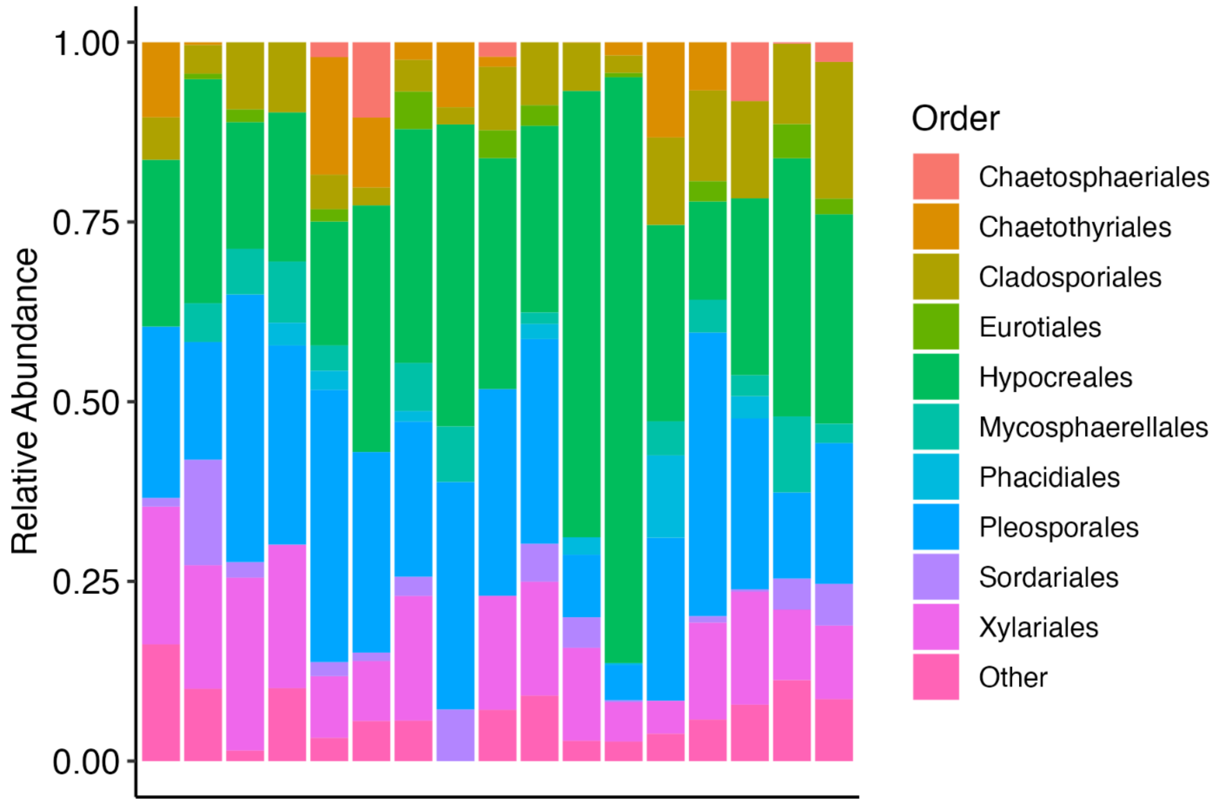

Supplemental Figure 3. Relative abundances of the top ten fungal orders in the rhizosphere inocula.

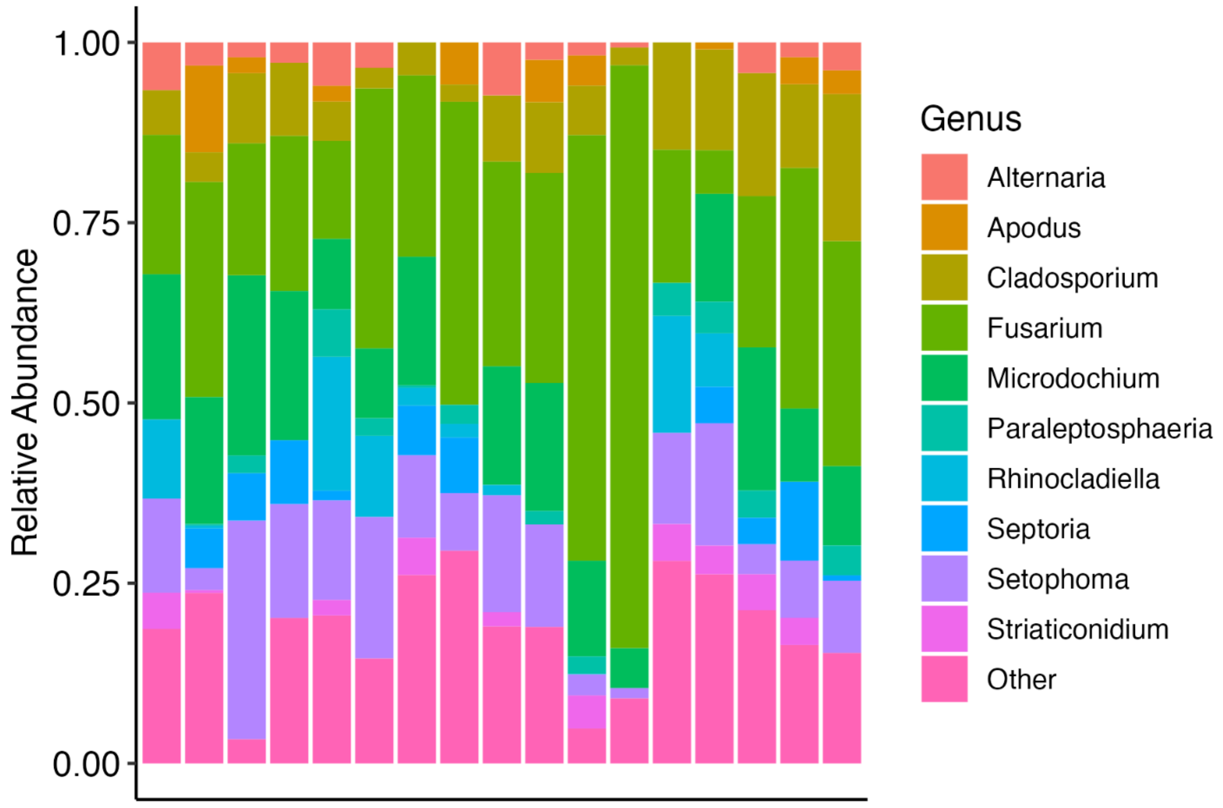

Supplemental figure 4. Relative abundances of the top ten fungal genera in the rhizosphere inocula.
